# Association between atypical endometriosis and ovarian malignancies in the real world

**DOI:** 10.1186/s13048-021-00865-2

**Published:** 2021-08-28

**Authors:** Kyeong A So, Sung Ran Hong, Nae Ri Kim, Eun Jung Yang, Seung-Hyuk Shim, Sun Joo Lee, Tae Jin Kim

**Affiliations:** 1 Department, of Obstetrics and Gynecology, Konkuk University School of Medicine, 120-1, Neungdong-ro, Gwangjin-gu, Seoul, 05030 Republic of Korea; 2 Department of Obstetrics and Gynecology, Cheil General Hospital & Women’s Healthcare Center, Seoul, Republic of Korea; 3 Department of Pathology, CHA Ilsan Medical Center, CHA University, Gyeonggi-do, Republic of Korea; 4 Department of Pathology, Cheil General Hospital & Women’s Healthcare Center, Seoul, Republic of Korea

**Keywords:** Atypical endometriosis, Ovarian malignancy, Clear cell carcinoma, Adenocarcinoma

## Abstract

**Background:**

To evaluate the clinical outcome of atypical endometriosis and its association with ovarian malignancy.

**Methods:**

This retrospective study included patients diagnosed with atypical endometriosis between January 2001 and December 2017. All patients had received surgical treatment for ovarian tumor. The clinical characteristics and histopathological results of all patients were reviewed.

**Results:**

Atypical endometriosis was diagnosed in 101 patients. We analyzed 98 patients with a mean age of 34.8 years (range: 16–58 years). Ten patients (10.2%) had previously undergone endometriosis surgery more than once. In total, 12 (12.2%) patients had atypical endometriosis-associated ovarian malignancy—nine had carcinomas and three had borderline tumor. The tumors were pathologically classified as follows: five, clear cell carcinomas; two, endometrioid adenocarcinomas; one, mixed clear cell and endometrioid adenocarcinoma; one, seromucinous carcinoma; two, mucinous borderline tumors; and one, seromucinous borderline tumor.

**Conclusion:**

Atypical endometriosis is most frequently associated with clear cell carcinoma and endometrioid adenocarcinoma. To identify the risk of ovarian malignancy and manage patients with endometriosis, diagnosing atypical endometriosis and recognizing its precancerous potential are important.

## Background

Endometriosis is defined as the presence of endometrial tissue outside the uterus [23]. It is a common gynecological disease that is estimated to occur in 5–8% of reproductive-aged women [20]. Although endometriosis is considered as a benign disease, it has a unique ability to metastasize and invade other tissues [27]. Some cases of endometriosis are closely related to ovarian malignancy [27]. In 1925, Sampson first described the criteria to diagnose a malignant tumor arising from endometriosis, which were as follows: evidence of endometriosis close to the tumor, exclusion of invasion from other sources, and presence of tissue resembling endometrial stoma surrounding characteristic epithelial glands [17]. In addition, in 1953, Scott indicated that transition of endometriosis from benign to malignant should be confirmed histologically [19].

Atypical endometriosis was first described by Czernobilsky and Morris in 1979 [3]. Atypical endometriosis is considered to have premalignant potential, is characterized by dysplastic features with cellular atypia, and is different from typical endometriosis [10]. Endometriosis-associated tumorigenesis involves pathophysiological progression of endometriosis to atypical endometriosis, followed by formation of a well-defined borderline tumor and development of ovarian malignancy [23]. However, it is difficult to diagnose accurately the atypical endometriosis. In the real world, there are few cases diagnosed as atypical endometriosis, so clinicians do not fully understand the clinical significance of atypical endometriosis. In this study, we investigated the clinical feature of atypical endometriosis and the association between atypical endometriosis and endometriosis-associated ovarian malignancy.

## Methods

This retrospective study included patients diagnosed with atypical endometriosis at Cheil General Hospital & Women’s Healthcare Center between January 2001 and December 2017. All patients had undergone surgical treatment for ovarian mass and were diagnosed with atypical endometriosis. Patients with a history of malignant disease were excluded. Medical charts were reviewed for clinical characteristics and laboratory findings. Further, histopathological results were reviewed by an expert gynecologic pathologist. The study was approved by the Institutional Review Board (No. CGH-IRB-2018–22). All procedures performed in this study were in accordance with the ethical standards of the institution and with the 1964 Helsinki declaration and its later amendments.

## Statistical analyses

Categorical variables are presented as numbers and percentages, and continuous variables are presented as means and standard deviations. Continuous and categorical variables were analyzed using a t-test and chi-square test, respectively. A *p*-value of less than 0.05 was considered statistically significant. Data analyses were performed using SPSS for Windows (version 17.0; SPSS Inc., Chicago, IL, USA).

## Results

A total of 101 patients were diagnosed with atypical endometriosis in the study period. Among them, two patients had endometrial adenocarcinoma and one patient had received treatment for ovarian mucinous borderline tumor 10 years ago. These three patients were excluded and remaining 98 patients were included in the study. During the same period, 13,074 patients were surgically diagnosed with endometriosis in this hospital. Therefore, the prevalence of atypical endometriosis was approximately 0.8%.

Clinical characteristics of the patients with atypical endometriosis are presented in Table 1. The mean age of patients was 34.8 years. In total, 88 (89.8%) patients were diagnosed with atypical endometriosis during their first surgery for endometriosis. Ten patients (10.2%) had previously undergone endometriosis surgery more than once. The mean duration from first surgery to diagnosis of atypical endometriosis was 7.4 years (range: 3–24 years). One patient was diagnosed with atypical endometriosis during the third surgery for ovarian endometriosis. The mean diameter of the ovarian cyst was 7.2 cm (range: 2–15.5 cm). Atypical endometriosis was found in the right ovary (55.1%), left ovary (35.7%), both ovaries (8.2%), and pelvic peritoneum (1%). Serum tumor markers were found to be elevated (CA-125: 87.0 U/mL, CA-19–9: 58.0 U/mL). Most of the atypical endometriosis cases were found to be benign endometriosis (87.7%); however, twelve cases (12.3%) of atypical endometriosis were found to be associated with malignant ovarian tumor.

**Table 1:** Patient characteristics of atypical endometriosis (*N* = 98)

Category	Number
Age (years, range)	34.8 ± 7.3 years (16 ~ 58 years)
Parity (range)	0.5 ± 0.9 (0 ~ 3)
Infertility history	17 (17.3%)
Number of previous surgery for endometriosis	
0	88 (89.8%)
1	9 (9.2%)
2	1 (1.0%)
Largest diameter (cm, range)	7.2 ± 2.7 (2 ~ 15.5)
Tumor marker (U/mL)	
CA-125	87.0 ± 100.2
CA-19–9	58.0 ± 60.2
Location of atypical endometriosis	
Right ovary	54 (55.1%)
Left ovary	35 (35.7%)
Both ovaries	8 (8.2%)
Pelvic peritoneum	1 (1.0%)
Histopathology	
Atypical endometriosis (AE)	86 (87.7%)
AE + Borderline tumor	3 (3.1%)
AE + Carcinoma	9 (9.2%)

Characteristics of atypical endometriosis with ovarian malignancy are presented in Table 2. The mean age of patients was 36.3 years and the mean diameter of the cyst was 8.8 cm (1.2–15 cm) in atypical endometriosis with ovarian malignancy. Malignant tumors associated with atypical endometriosis were borderline tumors (three cases) and invasive carcinoma (nine cases). These tumors were pathologically classified as follows: five, clear cell carcinoma (stage Ic); two, endometrioid adenocarcinoma (stage Ib and Ic); one, seromucinous carcinoma (stage IIb); and one, mixed carcinoma (clear cell and endometrioid; stage IIb).

**Table 2:** Characteristics of atypical endometriosis with ovarian malignancy (*N* = 12)

Category	Number
Age (years, range)	36.3 ± 5.2 (29 ~ 46)
Parity (range)	0.8 ± 1.0 (0 ~ 2)
Tumor size (cm, range)	8.8 ± 3.1 (1.2 ~ 15)
Tumor marker (U/mL)	
CA-125	122.9 ± 113.1
CA-19–9	43.8 ± 65.3
Location of atypical endometriosis	
Right ovary	6
Left ovary	6
Histopathology	
Borderline malignancy (*n* = 3)	Mucinous (*n* = 2), Seromucinous (*n* = 1)
Invasive carcinoma (*n* = 9)	
Clear cell carcinoma (5)	Stage Ic
Endometrioid adenocarcinoma (2)	Stage Ib, Ic
Mixed (clear cell + endometrioid) (1)	Stage IIb
Seromucinous carcinoma (1)	Stage IIb

The mean follow-up period for atypical endometriosis, except for patients with atypical endometriosis-associated ovarian malignancy, was 44.5 months. Seven of the 12 patients who were diagnosed with recurrent ovarian endometriosis by ultrasound underwent additional surgery. Two patients were diagnosed with recurrent atypical endometriosis. Two patients were diagnosed with recurrent atypical endometriosis after 4 years of the first surgery. One of these two patients had received gonadotropin-releasing hormone agonist treatment for 6 months after the first surgery, whereas the other did not receive any hormonal therapy after the first surgery

Comparison between atypical endometriosis alone and atypical endometriosis with ovarian malignancy is presented in Table 3. The mean size of ovarian cyst in atypical endometriosis with malignant tumor was significantly larger than that in atypical endometriosis alone (*p* = 0.025). No statistical differences in mean age, parity, and serum tumor maker levels were noted between the groups.

**Table 3:** Comparison between atypical endometriosis (AE) alone and AE with ovarian malignancy

Category	AE alone (*n* = 86)	AE + ovarian malignancy (*n* = 12)	*p-value*
Age (years)	34.6 ± 7.5	36.3 ± 5.2	0.452
Parity	0.5 ± 0.8	0.8 ± 1.0	0.344
Tumor size (cm)	7.0 ± 2.6	8.8 ± 3.1	0.025
Tumor marker (U/mL)			
CA-125	97.4 ± 13.0	122.9 ± 113.1	0.222
CA-19–9	43.8 ± 65.3	43.8 ± 65.3	0.464

## Discussion

Previous studies have reported that ovarian endometriosis may develop into primary malignant ovarian tumor. The risk of malignant transformation of endometriosis occurs in 0.7–4.5% of patients [1, 7, 21]. Endometriosis is associated with an increased risk of epithelial ovarian cancer development, especially clear cell and endometrioid carcinoma. Although clear cell and endometrioid subtypes are rare among all epithelial ovarian cancers, they are most common in endometriosis-associated ovarian cancer (EAOC) [8, 9, 16]. In our study, clear cell and endometrioid subtypes were found in 8 of 9 patients with invasive carcinoma. Clear cell carcinoma was the most common and was found in six patients. Several studies have indicated that patients with EAOC are more likely to be younger and to have longer disease-free survival than those with epithelial ovarian carcinoma not associated with endometriosis [2, 22]. In addition, EAOC is more likely to be diagnosed at an early stage and with low grade of tumor [6, 9, 12].

Incidence of ovarian atypical endometriosis is not well known. Two studies reported the incidence of atypical endometriosis to be approximately 1.7% and 5.9% in 255 and 339 benign ovarian endometriosis cases, respectively [5, 15]. The incidence of atypical endometriosis is found to be high (61–78%) in EAOC [5, 14]. In our study, the incidence of atypical endometriosis was approximately 0.8% in 13,074 patients diagnosed with benign endometriosis. The frequency of atypical endometriosis was low in our study because our study included considerably more cases than the previous studies. In addition, atypical endometriosis was diagnosed by extensive sections of specimen in previous studies. However, in real clinical practice, performing extensive pathological examination for each specimen is difficult. Therefore the real incidence of atypical endometriosis may be higher than 0.8% in the present study.

Atypical endometriosis represents a transition from endometriosis to carcinoma that may occur in the process of endometriotic tissue undergoing chronic inflammation and oxidative stress [11]. Several molecular and genetic mechanisms have been demonstrated to support that endometriosis may lead to epithelial ovarian carcinoma. EAOC is associated with overexpression of vascular endothelial growth factor (VEGF), and VEGF expression in atypical endometriosis may be associated with the malignant transformation of endometriosis [4]. Somatic mutations such as *ARID1A*, *PTEN*, and *PIK3CA* have been reported in patients displaying atypical endometriosis and development of endometrioid and clear cell carcinomas [13, 18, 25]. Activation of oncogenic KRAS and PIK3CA pathways and inactivation of tumor suppressor genes, *ARID1A* and *PTEN*, are observed in clear cell and endometrioid ovarian carcinomas, respectively [26].

The large size of atypical endometriotic cyst was associated ovarian malignancy (*p* = 0.025) in our study. Serum tumor marker levels did not distinguish patients with malignant ovarian tumors associated with atypical endometriosis. The risk of ovarian malignancy in patients with atypical endometriosis was not completely understood because of small sample size. However, atypical endometriosis has been implicated in the development of ovarian malignant tumor—a small number of patients will progress along the continuum from endometriosis to EAOC. It is not necessary for patients with endometriosis to be screened for cancer. However, if they are diagnosed with atypical endometriosis, they should be counseled for the potential risk of progression to endometriosis-associated ovarian malignancy [24].

## Conclusions

Diagnosing atypical endometriosis and recognizing its precancerous potential are important for identifying the risk of ovarian malignancy and managing patients with endometriosis. Results showed that the large size of atypical endometriotic cyst was associated ovarian malignancy. Further, careful long-term follow-up of the patients with large atypical endometriosis is required.

## Data Availability

The datasets used and/or analyzed in the present study are available from the corresponding author on reasonable request.

## Abbreviations

EAOC: Endometriosis-associated ovarian cancer
VEGF: Vascular endothelial growth factor
